# Rehabilitación de un caso con periodontitis activa y periimplantitis avanzada. Enfoque mínimamente invasivo

**DOI:** 10.21142/2523-2754-1002-2022-113

**Published:** 2022-06-27

**Authors:** Eduardo Anitua

**Affiliations:** 1 Práctica privada en implantología oral, Instituto Eduardo Anitua. Vitoria, España. eduardo@fundacioneduardoanitua.org Práctica privada en implantología oral Instituto Eduardo Anitua Vitoria España eduardo@fundacioneduardoanitua.org; 2 Investigador clínico, Fundación Eduardo Anitua. Vitoria, España. Investigador clínico Fundación Eduardo Anitua Vitoria España; 3 University Institute for Regenerative Medicine and Oral Implantology - UIRMI (UPV/EHU-Fundación Eduardo Anitua). Vitoria, España. University Institute for Regenerative Medicine and Oral Implantology - UIRMI (UPV/EHU-Fundación Eduardo Anitua) Vitoria España

**Keywords:** periodontitis, periimplantitis, periodontitis, periimplantitis

## Abstract

La enfermedad periodontal (EP) es una patología oral con una elevada prevalencia mundial. Existen diferentes tratamientos para su abordaje y la tendencia en los últimos años se dirige hacia los mínimamente invasivos. Cuando los implantes no pueden ser tratados de forma predecible a largo plazo, debemos plantearnos, además, la eliminación del implante con métodos atraumáticos y realizar un retratamiento del caso. Un punto a tener en cuenta tanto para la inserción de un implante dental como para su mantenimiento una vez afectado por periimplantitis, o para su recambio, es el estado periodontal del paciente. La enfermedad periodontal puede afectar el pronóstico de nuestro tratamiento en este tipo de situaciones. En el siguiente caso clínico, mostramos el tratamiento de un paciente afectado por EP y periimplantitis con un abordaje mínimamente invasivo.

## Abstract (en)

Periodontal disease (PD) is an oral pathology with a high prevalence worldwide. There are different treatments for periodontal disease, with the trend in recent years being towards minimally invasive treatments. In cases where implants cannot be treated predictably in the long term, we should also consider removing the implant using atraumatic methods and retreatment of the case. A point to be taken into account both for the insertion of a dental implant and for its maintenance after periimplantitis or for its replacement is the periodontal status of the patient. Periodontal disease can affect the prognosis of our treatment in this type of situation. In the following clinical case, we show the treatment of a patient affected by PD and periimplantitis with a minimally invasive approach.

## INTRODUCCIÓN

La enfermedad periodontal (EP) es una patología oral con una elevada prevalencia en la población mundial y es considerada por algunos autores como la infección crónica más común en adultos ^1^^-^^3^. Al igual que la caries, la enfermedad periodontal es una patología multifactorial en la que las bacterias juegan un rol importante, pero otros factores se encuentran implicados en su génesis y su desarrollo posterior. La mayoría de las bacterias relacionadas con el desarrollo de la EP son anaerobias ^4^^-^^5^ y, por ello, los procedimientos tradicionales para el tratamiento de esta patología buscan el control y disminución de este tipo de flora residente oral, así como la actuación sobre otros factores modificables que actúan en la patología (tabaco, hábitos de higiene oral y control de enfermedades asociadas bidireccionalmente, como la diabetes) ^4^^-^^9^. Sin embargo, en muchos de los pacientes que sufren EP, pese a la realización de numerosos tratamientos, se produce la pérdida de los dientes afectados. 

Para realizar un correcto diagnóstico del paciente periodontal y tomar decisiones sobre el mantenimiento a largo plazo de sus piezas dentales, debemos tener en cuenta los factores predictivos que se han descrito para la pérdida dental en estos casos. Dichos factores se clasifican usualmente como factores a nivel del paciente (sexo, edad y determinados polimorfismos genéticos, como los de la IL-1), que lo hacen más susceptible, como aquellos factores a nivel diente (estado del diente, movilidad, sondaje y afectación de la furca) ^10^^-^^12^. Con estos factores en mente, y otros relacionados con la decisión del paciente en cuanto a conservar sus dientes y a implicarse activamente con el tratamiento de mantenimiento -o la opción de pasar a un tratamiento más agresivo según las necesidades y prioridades-, debemos abordar a nuestro paciente periodontal como un todo, a la vez que generamos un tratamiento individualizado. Mantener o extraer determinados dientes es, por lo tanto, una opción compleja que debe englobar diferentes puntos en el diagnóstico y en el pronóstico de los dientes valorados, y se trata siempre de una disyuntiva importante a la hora de tratar este tipo de pacientes ^13^.

La utilización de implantes dentales en pacientes afectados de EP es un hecho hoy en día extendido en la práctica odontológica. En algunos de los pacientes, vemos convivir implantes dentales con dientes afectados por EP e, incluso, estos implantes pueden ser insertados en localizaciones donde se ha perdido el diente por esta patología o colocarlos en zonas donde se ha perdido ya un implante dental afectado por periimplantitis. La relación entre estas dos patologías ha sido discutida durante mucho tiempo, y se ha establecido en trabajos recientes una mayor probabilidad de desarrollo de periimplantitis en pacientes con EP previa ^14^. Aun así, existen diferencias entre ambas patologías, como la progresión de la enfermedad, la respuesta al tratamiento a largo plazo (peor para la periimplantitis, con tasas de recurrencia de hasta el 100% en algunos estudios en los siguientes 12 meses de seguimiento) y las bacterias implicadas ^15^. La realidad es que, en la clínica dental, la concurrencia de las dos patologías en pacientes existe y, en ocasiones, tenemos que abordar pacientes con periodontitis y periimplantitis, así como realizar un abordaje de ambas patologías en busca de la mejor opción para el paciente a partir de la predictibilidad del tratamiento ^16^^,^^17^. En el siguiente caso clínico, mostramos el tratamiento de un paciente afectado por EP y periimplantitis con un abordaje mínimamente invasivo. 

### Caso clínico

**Figura 1:**
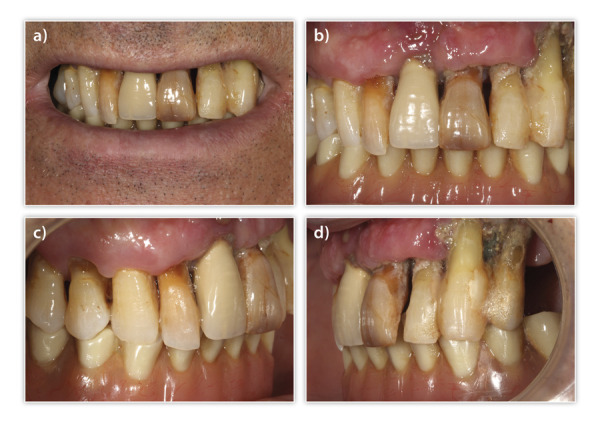
A-B) Imágenes intraorales iniciales del paciente donde ya puede verse la enfermedad periodontal avanzada que presenta. C-D) En las imágenes laterales, podemos observar las recesiones múltiples con una recesión ósea completa en las piezas del segundo cuadrante.

**Figura 2:**
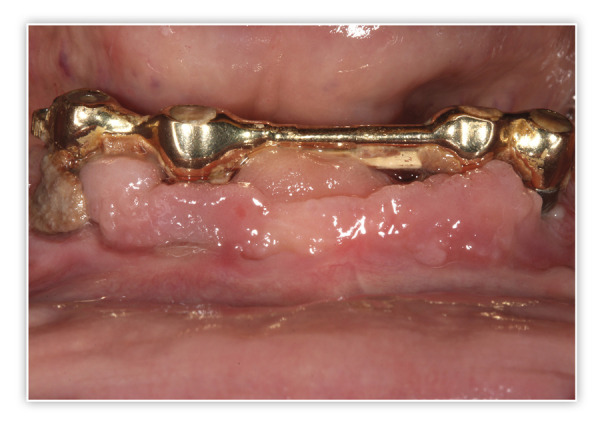
Imagen de la zona de la barra de la sobredentadura. Podemos observar la acumulación de placa bacteriana sobre las superficies de los implantes expuestos, la supuración espontánea en otros de ellos y gran inflamación.

**Figura 3:**
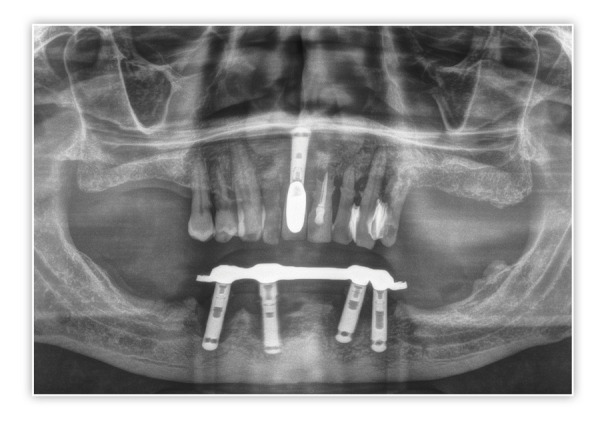
Radiografía panorámica inicial donde podemos observar los defectos en cráter de los implantes, así como los defectos horizontales de los dientes remanentes.

Presentamos el caso de un paciente varón, de 60 años, con una enfermedad periodontal avanzada que le causa dolor, halitosis y tiene repercusión en su día a día, además de un problema de dolor, movilidad y supuración en unos implantes inferiores que le colocaron hacer varios años para instalar una sobredentadura. En la exploración inicial, podemos ver una gran pérdida ósea horizontal que ha dejado expuestas todas las raíces dentales con zonas donde el defecto es mayor, como el segundo cuadrante, por lo que las raíces quedan expuestas casi al completo (Figura 1). Al retirar la sobredentadura, observamos, además, inflamación y supuración de los tejidos blandos, así como acumulación de placa bacteriana en zonas donde el implante se encuentra expuesto al medio oral (Figura 2). Como primer paso de la planificación, se lleva a cabo una OPG en la que se evidencian pérdidas óseas circunferenciales en cráter en los implantes y las pérdidas óseas dentales, que son incluso mayores de lo que parecía en la exploración intraoral en los dientes anteriores del segundo cuadrante que no presentan tanta recesión (Figura 3). 

**Figura 4:**
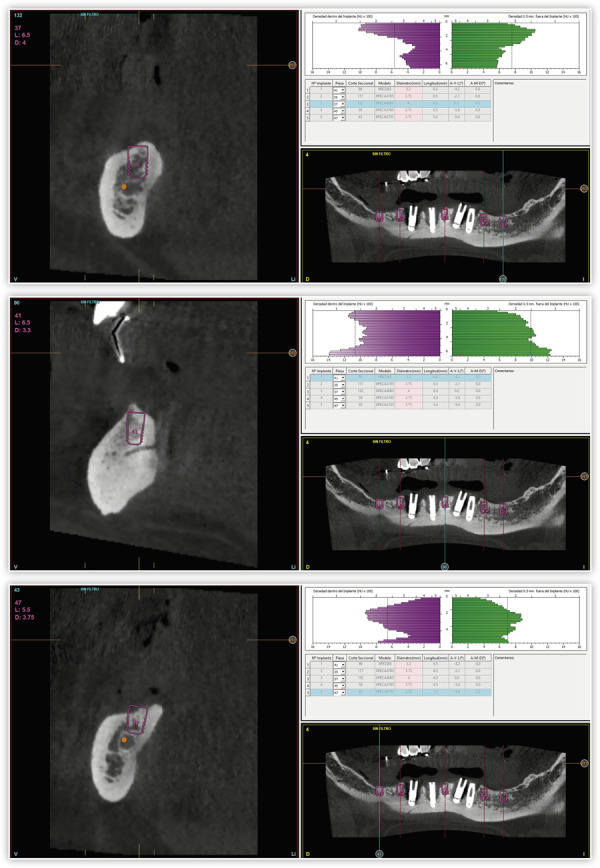
Imágenes del cone-beam de planificación.

**Figura 5:**
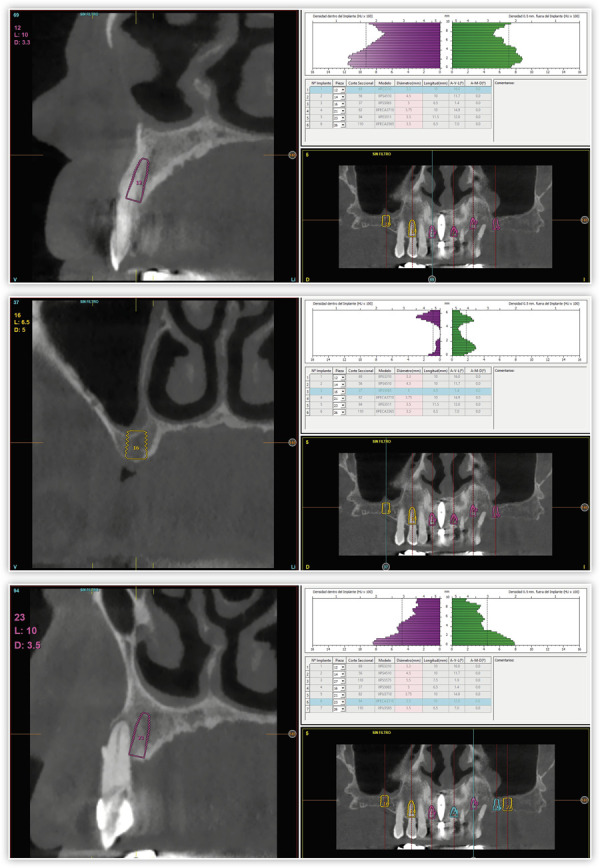
Planificación de la inserción directa de implantes en sectores posteriores de forma directa.

**Figura 6:**
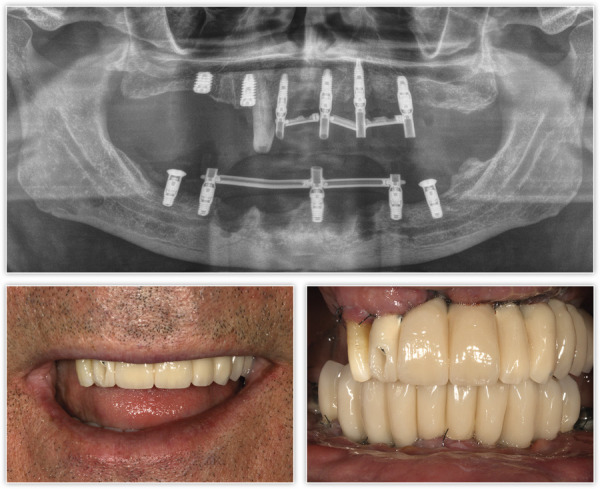
Radiografía e imágenes de la prótesis de carga inmediata en el momento de su colocación.

Para continuar con el tratamiento, se llevó a cabo un *cone-beam* que permitía diagnosticar las explantaciones y la nueva inserción de los implantes que reemplazarán a los afectados por periimplantitis. En la planificación, constatamos que era posible insertar implantes de forma directa en ambos sectores posteriores por detrás del último implante actual (dos implantes en cada cuadrante), así como un implante central (Figura 4). En el caso del maxilar superior, se planificó la extracción de todas las piezas dentales, con excepción de un canino superior derecho que de momento nos servirá para mantener la propiocepción en los momentos iniciales de los provisionales sobre implantes. Posteriormente, se valorará la posible conservación del diente una vez finalizado el tratamiento en función de su estabilidad periodontal. En la misma cirugía se realizó la extracción de las piezas dentales, así como la inserción de los implantes, algunos en los lechos posextracción de los dientes tras un legrado minucioso del alveolo para retirar todo el tejido inflamatorio (Figura 5). Una vez finalizada la cirugía superior e inferior, se procedió a la carga inmediata de los implantes con excepción de dos del primer cuadrante y dos distales en ambos extremos mandibulares que no han alcanzado la estabilidad primaria suficiente para englobarse en ella (menos de 20 Ncm). El resto se cargaron en 24 horas con una prótesis elaborada mediante barras articuladas (Figura 6). Una vez regenerados los defectos remanentes en los que no podían insertarse los implantes de forma directa con PRGF-Endoret, se llevó a cabo un segundo abordaje en el que se terminarán de colocar los implantes necesarios para la rehabilitación final. 

**Figura 7:**
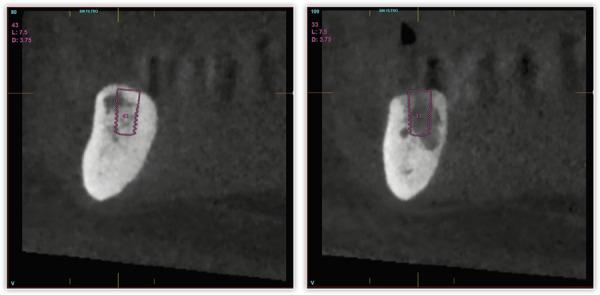
Cortes seccionales de las zonas regeneradas previos a la inserción de los implantes mandibulares.

**Figura 8:**
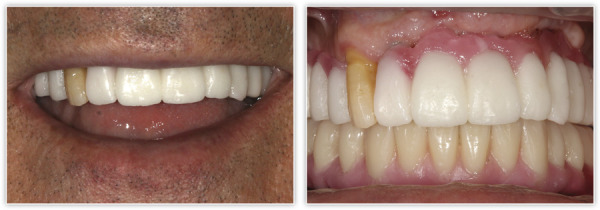
Imágenes de la prueba de ambos encerados.

**Figura 9:**
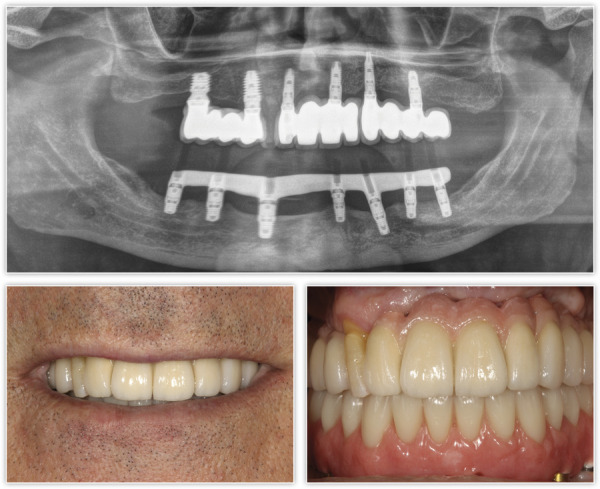
Imagen radiográfica final de ambas prótesis colocadas: puentes atornillados sobre transepitelial metal-cerámica por sectores y prótesis híbrida inferior.

**Figura 10:**
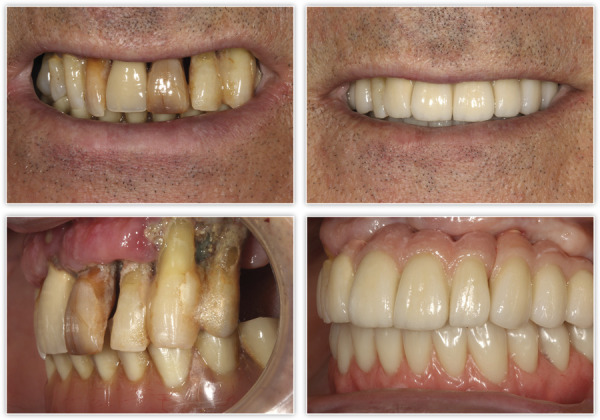
Comparativa inicial y final de la evolución del caso clínico, y resolución de los problemas estéticos, funcionales y de salud periodontal.

**Figura 11:**
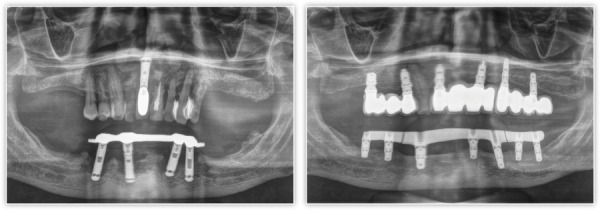
Radiografía inicial y final del paciente tras el tiempo de seguimiento.

Tras dos meses de la cirugía inicial, se procedió a la inserción de los implantes inferiores que no pudieron colocarse en la primera intervención debido a los defectos óseos generados por la periimplantitis. La regeneración del defecto se ha completado, como se puede ver, en los cortes seccionales de planificación empleando únicamente PRGF-Endoret (Figura 7). Una vez transcurrido el período de integración de ambos implantes (tres meses), se empezó a confeccionar la prótesis definitiva. Finalmente, el canino en posición 13 ganó inserción y se decidió conservarlo. Se construyó un encerado a partir de la prótesis provisional para ser probado en boca y generar así los cambios necesarios a nivel estético y oclusal (Figura 8). La prótesis final se realizó en metal-cerámica atornillada a transepitelial por sectores en el arco superior y una híbrida inferior atornillada sobre transepiteliales (Figura 9). Con esta rehabilitación se ha logrado recuperar la estética, función y salud que se había perdido con el progreso de la EP y la periimplantitis, tal como podemos observar en las imágenes comparativas antes-después (Figura 10) y en la comparativa radiológica inicial y a los tres años (Figura 11).

## DISCUSIÓN

La periimplantitis es un problema que debemos abordar de forma rutinaria en la consulta dental, pues se trata de una patología destructiva que nos obliga a realizar retratamientos en pacientes que han perdido un volumen óseo debido al curso de la enfermedad y que en ocasiones limita nuestro rango de acción ^18^^,^^19^. La conservación de los implantes afectados por periimplantitis es un hecho controvertido, ya que en muchas ocasiones la limpieza de la superficie colonizada por las bacterias es muy compleja; por ello, lograr una estabilización del cuadro y frenar la progresión de la destrucción ósea una vez realizado el tratamiento no es posible ^20^^,^^21^. En casos avanzados como el que se muestra en el presente artículo, la posibilidad de revertir la patología y lograr detoxificar la superficie del implante no es una opción, por lo que la explantación de forma atraumática, mediante el kit de explantación (KEXIM) utilizado por nuestro grupo de estudio en publicaciones previas es la mejor forma de abordar el caso ^22^^-^^25^. Una vez extraídos los implantes y regeneradas las zonas con mayor defecto óseo podemos iniciar una nueva rehabilitación con implantes dentales. Incluso, en las zonas donde la pérdida ósea lo permite, la inserción de un nuevo implante en el mismo lecho posexplantación es posible, debido a que el kit de extracción de implantes permite conservar íntegro el lecho óseo ^26^^,^^27^. La predectibilidad de este implante recambiado en el mismo lecho se encuentra también documentada, y no se ha reportado una mayor tasa de fracaso que la de un implante insertado en una zona donde no existiese previamente esta patología, siempre que se realice de forma correcta la retirada del implante y la limpieza del alveolo posexplantación, para eliminar todo el tejido inflamatorio ^26^^,^^27^. 

En este caso clínico hemos mostrado, además, la presencia de una EP avanzada y activa en el momento de la extracción dental y la colocación de nuevos implantes. Aun en este caso más extremo, se ha logrado realizar la carga inmediata en implantes que podemos denominar claves para mantener al paciente en todo momento con dientes fijos. La supervivencia media de los implantes insertados posextracción con carga inmediata se encuentra en un 98,4% (después de 2 años) y desciende hasta un 97,5% (rango 95,2-98,8% tras 3 años de seguimiento) ^28^. Las cifras de supervivencia son elevadas; por lo tanto, no debemos tener reparos a la hora de realizar este tipo de técnica siempre y cuando, al igual que en la zona de los implantes antes mencionados, el protocolo de inserción y desbridamiento de los tejidos blandos residuales en los alveolos sea llevado a cabo de forma cuidadosa ^29^.

El abordaje de estos casos con patologías inflamatorias crónicas de forma mínimamente invasiva para reducir el número de intervenciones (implantes inmediatos posexodoncia y posexplantación) mejora la situación clínica del paciente de forma rápida, ya que mantiene, en la medida de lo posible, al paciente con una prótesis fija en todo momento, tal como lo hemos mostrado en este caso ^30^^-^^31^. 

## CONCLUSIONES

El abordaje de los pacientes con enfermedad periodontal y periimplantitis, con defectos óseos que hacen imposible la conservación de dientes e implantes, debe ser tenido en cuenta desde un punto de vista restaurativo y quirúrgico, a fin de evitar técnicas complejas regenerativas que acumulen una mayor probabilidad de fracaso. La inserción inmediata de implantes tanto en zonas de extracción como de explantación, y la carga inmediata de estos, no supone un factor crítico siempre y cuando los protocolos quirúrgicos y protésicos se encuentren individualizados y basados en evidencia científica.
